# Discovery of skill-switching criteria for learning agile quadruped locomotion

**DOI:** 10.3389/frobt.2026.1697159

**Published:** 2026-02-18

**Authors:** Wanming Yu, Fernando Acero, Vassil Atanassov, Chuanyu Yang, Ioannis Havoutis, Dimitrios Kanoulas, Zhibin Li

**Affiliations:** 1 Department of Engineering Science, Oxford Robotics Institute, University of Oxford, Oxford, United Kingdom; 2 Department of Computer Science, University College London, London, United Kingdom; 3 National Elite Institute of Engineering, Chongqing University, Chongqing, China

**Keywords:** deep reinforcement learning, gait transitions, hierarchical learning and optimization, legged locomotion, multi-skill locomotion, robot learning, skill switching

## Abstract

This study develops a hierarchical learning and optimization framework that can learn and achieve well-coordinated multi-skill locomotion. The learned multi-skill policy can switch between skills automatically and naturally while tracking arbitrarily positioned goals and can recover from failures promptly. The proposed framework is composed of a deep reinforcement learning process and an optimization process. First, the contact pattern is incorporated into the reward terms to learn different types of gaits as separate policies without the need for any other references. Then, a higher-level policy is learned to generate weights for individual policies to compose multi-skill locomotion in a goal-tracking task setting. Skills are automatically and naturally switched according to the distance to the goal. The appropriate distances for skill switching are incorporated into the reward calculation for learning the high-level policy and are updated by an outer optimization loop as learning progresses. We first demonstrate successful multi-skill locomotion in comprehensive tasks on a simulated Unitree A1 quadruped robot. We also deploy the learned policy in the real world, showcasing trotting, bounding, galloping, and their natural transitions as the goal position changes. Moreover, the learned policy can react to unexpected failures at any time, perform prompt recovery, and successfully resume locomotion. Compared to baselines, our proposed approach achieves all the learned agile skills with improved learning performance, enabling smoother and more continuous skill transitions.

## 1 Introduction

**FIGURE 1:**
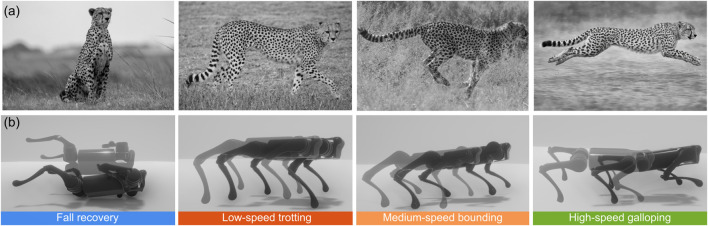
Coordination and gait transitions in quadrupedal animal and robot under increasing speed demands. **(a)** Cheetah’s changing gaits at increasing speed. **(b)** *A1* quadruped robot’s fall recovery, trotting, bounding, and galloping skills using our multi-skill policy. Images in (a) were adapted from https://unsplash.com/photos/cheetah-walking-on-green-grass-field-during-daytime-0SsN7jfCXps, https://unsplash.com/photos/cheetah-walking-on- brown-grass-field-during-daytime-RRLEw1yCbe0 and https://unsplash.com/photos/brown-and-black-jaguar-zbnOYJo6mKc, respectively. The last image was generated by ChatGPT.

Animals have evolved highly efficient movement strategies. Mimicking these can improve legged locomotion in terms of agility, stability, and adaptivity (Figure 1). In particular, animals learn to switch between motor skills swiftly according to tasks and surroundings. For instance, horses switch to different gait patterns as the speed changes (Hoyt and Taylor, 1981). However, reproducing multiple gaits and their dynamically feasible transitions on legged robots remains challenging in the robot learning and control community. In addition, the ability to recover from various failures, which is of vital interest for successful and resilient real-world deployment, is not yet well-studied in multi-skill locomotion. A multi-skill framework has the ability to recover from failures during locomotion (Yang et al., 2020; Yuan and Li, 2022); however, it does not show more dynamic gaits beyond trotting. Although improving robustness against failures or fall recovery has been studied in several previous works (Hwangbo et al., 2019; Castano et al., 2019; Cordie et al., 2024), it is learned as a single skill and cannot be combined with other skills.

### 1.1 Reproducing gait patterns

Behavior cloning or imitation learning approaches have been applied to reproduce various gaits from reference motions. Reference motions can be captured from animal locomotion, which may be limited in variety or generated using model predictive controllers (Reske et al., 2021), which require domain knowledge and computational efficiency. In general, such approaches can be difficult to scale beyond the dataset, thereby lacking robustness and diversity of the learned behaviors. There have been attempts to model or learn parameterized control policies to achieve different styles of walking. A phase-guided controller is used to learn gait transitions between walking, trotting, pacing, and bounding on a quadrupedal robot (Shao et al., 2021). A single policy is learned to control various gaits with variable footswing, posture, and speed (Margolis and Agrawal, 2023). Simple balance control has been inspired by a bicycle to achieve high-speed running on a quadruped robot (Hattori et al., 2025). However, such approaches require handcrafted, control-specific behavior parameters, which demand domain knowledge and are not intuitive for gait switching.

### 1.2 Generative models in multi-skill locomotion

Recent advances in generative models have achieved low-level control of locomotion gaits on quadrupedal robots. Variational autoencoders have been applied to learn a disentangled and two-dimensional latent representation across locomotion gaits with respect to footswing heights and lengths (Mitchell et al., 2024). Given the desired gait type and swing characteristics, it achieved low-level control of trotting, crawling, and pacing on quadrupedal robots. In addition, diffusion models have demonstrated the capability of achieving multi-skill locomotion control with a single policy, including trotting, hopping, pacing, walking, and running (Huang et al., 2024), along with walking, crawling, and their transitions (O’Mahoney et al., 2025). However, these generative models require expert demonstrations, and the performance is dependent on the quality of the dataset. Moreover, the gait type was conditioned on a certain input to the control policy. In contrast, gait types were autonomously discovered by our proposed hierarchical framework, which covers both high-level and low-level multi-skill locomotion control.

### 1.3 Foundation models in legged locomotion

Applying foundation models in robot learning applications is a favorable approach for achieving generalized robot tasks and behaviors. Pre-trained vision-language models (VLMs) usually focus on high-level reasoning and planning to select from a set of existing low-level skills (Chen et al., 2025). However, certain low-level locomotion skills can be difficult to obtain in practice. Several attempts have also been made to apply pre-trained large language models (LLMs) to achieve multiple gaits via low-level interfaces, such as foot contact patterns (Tang et al., 2023). In general, these foundation models in robotic applications require either careful prompt engineering or a huge amount of robotic data for fine-tuning. In practice, robotic data can be difficult to obtain in certain cases, and fine-tuning of large-scale models may require substantial computational resources.

### 1.4 Bio-inspired multi-gait locomotion

Unlike the above approaches requiring reference motions, some robotics research has applied deep reinforcement learning to acquire animal-like gait transitions based on various criteria inspired by biological principles, where reference motions are not necessary. By minimizing energy consumption (Miranda et al., 2025), the robot can achieve gait transitions between walking, trotting, and fly-trotting at different speed ranges using a single policy (Liang et al., 2025) or a hierarchical structure (Yang et al., 2022), along with gait transitions from walking to trotting to bouncing (Fu et al., 2021). Another bio-inspired research modulates gait transitions according to Froude numbers (Humphreys et al., 2023). A more recent work learned gait transitions from walking to trotting on flat ground and trotting to pronking when crossing gaps according to viability (Shafiee et al., 2024). However, galloping cannot emerge or be incorporated at high speed on mechanical robots in the above frameworks. Moreover, a series of works utilizes central pattern generators (CPGs) to produce different gaits by deep reinforcement learning on quadrupedal robots. The most recent work adopted a coupling-driven approach to learn a policy via deep reinforcement learning to modulate the parameters of CPGs, producing nine gaits and transitions, including galloping, based on the cost of transport (CoT) (Bellegarda et al., 2025). In contrast with bio-inspired multi-gait locomotion, our proposed framework can produce a galloping gait. Moreover, our approach is not constrained by biological principles as we can define customized cost terms for optimizing gait switch timing. In addition to biology-inspired criteria, we can also include other cost terms, such as task-related costs.

### 1.5 State-of-the-art quadrupedal locomotion

A parallel line of research demonstrated impressive dynamic parkour skills in legged robots (Caluwaerts et al., 2023; Cheng et al., 2024; Zhuang et al., 2023; He et al., 2024). However, these works usually focused on navigating the robot along a series of challenging terrains and obstacles. In most cases, a simple goal-reaching task is considered in these navigation tasks, while gait patterns are not taken into account. In contrast, this study focuses on multi-skill navigation and control tasks, i.e., reaching arbitrary goals with various gait patterns and their transitions.

### 1.6 Contributions

To summarize, the advantages of our proposed approach over the existing literature include (1) we do not require reference trajectories or expert demonstrations, and our model can learn multi-skill locomotion purely from scratch; (2) animal-like galloping gait can be activated at high-speed locomotion; (3) autonomous fall recovery is incorporated in the multi-skill policy, enabling high robustness and requiring less human intervention; and (4) flexible gait-switch criteria are automatically discovered for mechanical robots. To the best of our knowledge, our work is the first multi-skill learning and optimization framework that is compatible with incorporating and synthesizing multiple highly dynamic locomotion gaits (especially galloping) and producing natural, dynamically feasible transitions by automatically discovered gait-switch criteria. Our work demonstrates four skills on a quadruped robot in the real world, including prompt fall recovery at any stage during multi-skill locomotion. To summarize, the contributions of this study include the following:

- Incorporating highly dynamic locomotion skills of bounding and galloping in addition to trotting into learning one coherent multi-skill policy, without the need for reference trajectories.
- Demonstrating successful trotting, bounding, and galloping and their dynamically feasible and continuous transitions with one synthesized multi-skill policy on a real quadruped robot.
- Successful failure recovery at any stage of different gaits.
- Automatic discovery of gait-switch criteria as motor learning progresses, which converges to a higher training reward faster than baselines.

**FIGURE 2:**
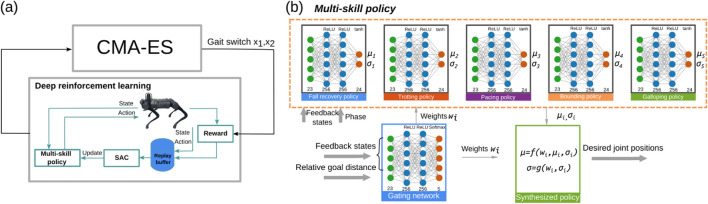
Proposed multi-skill learning and optimization framework. **(a)** Optimizing gait-switch criteria in the outer-loop of deep reinforcement learning. **(b)** Neural network architecture of a multi-skill policy. Bold arrows indicate the input or output outside the policy, while normal arrows indicate the internal input or output.

Our hierarchical multi-skill learning and optimization framework is shown in Figure 2, which includes (1) a set of pre-trained reusable single-skill neural network policies, each representing a single locomotion skill; (2) a task-level neural network that generates weights for each skill to produce our multiplicative composite policy; (3) the composite multi-skill policy; and (4) the outer optimization loop for the discovery of gait-switch criteria represented by the relative goal distances in the horizontal plane that activate the switch from trotting to bounding and the switch from bounding to galloping, respectively.

In the following sections, we first review the details of our hierarchical learning and optimization framework in Sections 2.1 and 2.2. Then, we demonstrate and analyze the learned multi-skill locomotion policy on a real quadruped robot in Section 3. Finally, we conclude our work in Section 4.

## 2 Materials and methods

### 2.1 Hierarchical multi-skill learning framework

#### 2.1.1 Learning individual skills

The robot learns five individual skills separately using a systematic deep reinforcement learning framework, namely, fall recovery, trotting, pacing, bounding, and galloping. Each locomotion skill is a feedback control policy represented by a neural network, which is learned using the Soft Actor–Critic (SAC) algorithm (Haarnoja et al., 2018). Details of the key components for our deep reinforcement learning framework are given below. Each individual skill is a fully connected neural network with two hidden layers. Each hidden layer has 256 neurons and uses a ReLU activation function, and the output layer uses a tanh activation function. The output of each neural network is 24-dimensional, including the mean and variance of all 12 joints. For skill i , $\mu_{i}^{j}$ and $\sigma_{i}^{j}$ represent the mean and variance of the j th joint, respectively, and the final desired position of the j th joint, $\hat{q_{i}^{j}}$ , is sampled from the corresponding Gaussian distribution $N\left(\mu_{i}^{j},\sigma_{i}^{j}\right)$ .

##### 2.1.1.1 State observation and action space

Following the key feedback states in learning locomotion skills (Yu et al., 2023), the state input to the actor neural network includes (1) normalized gravity vector in the robot local frame, which reflects the body orientation of the robot, (2) base angular velocity, (3) base linear velocity in the robot heading frame, and (4) joint positions. For learning periodic locomotion skills, we also included a two-dimensional phase vector (sin2πϕ,cos2πϕ) to represent continuous temporal information that encodes phase ϕ from 0% to 100% of a periodic motion. The actions are the desired joint positions for 12 joints, including hip roll, hip pitch, and the knee joints of four legs.

##### 2.1.1.2 Reward design

Trotting and bounding were learned with a fixed desired velocity, while galloping was learned by maximizing velocity. The reward function for learning individual policies is composed of continuous and discrete reward terms. For continuous reward terms, we use a radial basis function (RBF) to formulate as in Equation 1:

$$\varphi \left(x,\hat{x},\alpha \right)=\exp\left(\alpha {\left(\hat{x}-x\right)}^{2}\right),$$ (1)

where x is the continuous physical quantity, $\hat{x}$ is the corresponding reference, and α is the shape parameter that controls the width of RBF. The formulation and weight of each reward term are provided in Tables 1 and 2, respectively. There are 11 reward terms in total for training individual skills. The essential reward terms that distinguish different skills are the base linear velocity rewards (different desired velocity ranges for different gaits) and reference foot contact rewards (different contact patterns for different gaits; see Section 2.1.1.3). The remaining reward terms are commonly used in the legged locomotion field to maintain locomotion stability, such as preserving a certain orientation and height, minimizing energy consumption by reducing joint torques and velocities, and preventing falls by penalizing unintended body contacts with the ground while encouraging proper foot contacts.

**TABLE 1:** Reward terms for learning quadruped locomotion skills.

Physical quantity	Reward term
Base orientation	$w_{\phi }\times \varphi \left(\phi ,\left[0,0,-1\right],-2.35\right)$
Base height	$w_{h}\times \varphi \left(h,\hat{h},-51.16\right)$
Base linear velocity	$w_{v}\times \left\{\begin{array}{cc} {{v_{x}}_{\mathrm{base}}^{\mathrm{world}}}^{2},\; & \text{gallop}\\ \varphi \left(v_{\mathrm{base}}^{\mathrm{world}},{\hat{v}}_{\mathrm{base}}^{\mathrm{world}},-18.42\right),\; & \text{else} \end{array}\right.$
Joint torque	$w_{\tau }\times \varphi \left(\tau ,0,-0.004\right)$
Joint velocity	$w_{\dot{q}}\times \varphi \left(\dot{q},0,-0.032\right)$
Body–ground contact	$w_{bg}\times \left\{\begin{array}{cc} 0,\; & \text{base in contact with ground}\\ 1,\; & \text{base not in contact with ground} \end{array}\right.$
Foot–ground contact	$w_{fg}\times \left\{\begin{array}{cc} 0,\; & \text{no foot in contact with ground}\\ 1,\; & \text{foot in contact with ground} \end{array}\right.$
Symmetric foot placement	$w_{pf}\times \left\{\begin{array}{cc} \varphi \left(p_{\mathrm{foot}}^{\mathrm{base}},{\hat{p}}_{\mathrm{foot}}^{\mathrm{base}},-51.16\right),\; & \text{recovery}\\ \varphi \left(1/4\sum_{n=1}^{4}\left(p_{\mathrm{foot,n}}^{\mathrm{world}}\right),p_{\mathrm{base}}^{\mathrm{world}},-51.16\right),\; & \text{gaits} \end{array}\right.$
Swing and stance	$w_{hf}\times \varphi \left(h_{\mathrm{foot}}^{\mathrm{world}}v_{\mathrm{foot}}^{\mathrm{world}},{\hat{h}}_{\mathrm{foot}}^{\mathrm{world}}v_{\mathrm{foot}}^{\mathrm{world}},-460.50\right)$
Yaw velocity	$w_{\dot{\psi }}\times \varphi \left(\dot{\psi },0,-7.47\right)$
Reference foot contact	$w_{f}\times \left\{\begin{array}{cc} 0,\; & \text{not match desired foot contact}\\ 1,\; & \text{match desired foot contact} \end{array}\right.$

**TABLE 2:** Reward term weights for learning single locomotion skills.

Task	$w_{\phi }$	$w_{h}$	$w_{v}$	$w_{\tau }$	$w_{\dot{q}}$	$w_{bg}$	$w_{fg}$	$w_{pf}$	$w_{hf}$	$w_{\dot{\psi }}$	$w_{f}$
Fall recovery	0.189	0.189	0.114	0.076	0.076	0.083	0.083	0.189	0.000	0.000	0.000
Gait	0.068	0.068	0.170	0.017	0.017	0.048	0.000	0.034	0.034	0.068	0.476

##### 2.1.1.3 Reference foot-contact reward

**FIGURE 3:**
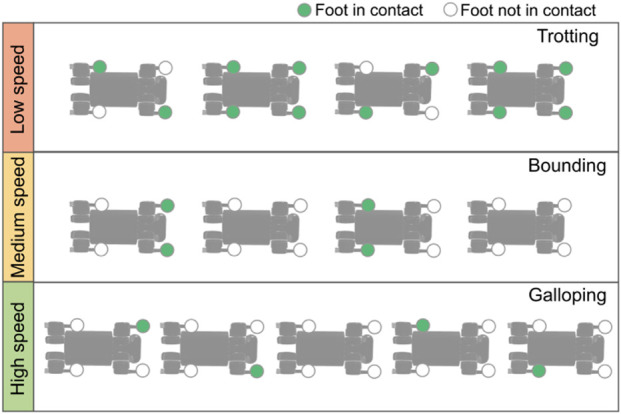
Foot-contact patterns across different speed ranges.

The last reward term in Table 1, i.e., the reference foot-contact reward, is the key to learning different gait types without reference. The desired foot-contact pattern for each gait type is inspired by quadrupedal animals (Owaki and Ishiguro, 2017), as shown in Figure 3. In this study, we assume trotting, bounding, and galloping gaits as speed increases, as a proof-of-concept. It should be noted that the order of gait types is not fixed. Here, we determine the gait type at different stages according to its characteristics.

#### 2.1.2 Learning multi-skill locomotion

The task objective of the robot is to track an arbitrary goal in the horizontal plane with natural gait transitions from trotting to bounding to galloping. The goal is represented by $\left(d_{g},\theta_{g}\right)$ , and the goal position in the horizontal plane is $\left(d_{g}cos\theta_{g},d_{g}sin\theta_{g}\right)$ . At each episode, we set a fixed goal, where $d_{g}∼\left(0m,15m\right)$ and $\theta_{g}∼\left(-180{}^{\circ},180{}^{\circ}\right)$ . The multi-skill locomotion policy is synthesized on-the-fly by the multiplicative composition (Peng et al., 2019) of the low-level pre-trained individual policies according to the output from the high-level gating network. We introduce more details about each component separately.

##### 2.1.2.1 Gating network

Our high-level gating network is a fully connected neural network with two hidden layers. Each hidden layer has 256 neurons and uses a ReLU activation function, while the output layer uses a Softmax function. The gating network receives the following as input: gravity vector, base angular velocity, base linear velocity, joint positions, and the normalized distance between the robot and the goal in the horizontal plane. The gating network outputs the weights for each locomotion skill, which add up to one.

##### 2.1.2.2 Composite multi-skill policy

During the training of multi-skill locomotion, the parameters of the expert networks are transferred from the pre-trained single-skill policies and remain fixed throughout. That is, only the gating network parameters are updated by backpropagation of the gradient obtained from the designed reward. The weights for each locomotion skill generated by the gating network are then applied to synthesize the multi-skill Gaussian policy π(a|s,g) by the multiplicative composition of n pre-trained single skills as in Equation 2:

$$\pi \left(a|s,g\right)=\frac{1}{Z\left(s,g\right)}\overset{n}{\underset{i=1}{\prod }}\pi_{i}{\left(a|s\right)}^{w_{i}\left(s,g\right)},\;w_{i}\left(s,g\right)\geq 0,$$ (2)

where $\pi_{i}\left(a|s\right)$ is the i th single-skill neural network policy, $w_{i}\left(s,g\right)$ is the weight for the corresponding skill to influence the composite policy, and Z(s,g) is the normalization factor. The synthesized policy is a multiplicative composition of n Gaussian policies, i.e., n single skills. As discussed by Peng et al. (2019), the multiplicative composition of Gaussian primitives results in another Gaussian policy, i.e., the composite policy. Due to the use of Gaussian primitives, the composite mean $\mu^{j}$ and composite variance $\sigma^{j}$ of the j th joint of the synthesized Gaussian policy are obtained as follows:

$$\mu^{j}\left(s,g\right)=\frac{1}{\sum_{i=1}^{n}\frac{w_{i}\left(s,g\right)}{\sigma_{i}^{j}\left(s,g\right)}}\overset{n}{\underset{i=1}{\sum }}\frac{w_{i}\left(s,g\right)}{\sigma_{i}^{j}\left(s,g\right)}\mu_{i}^{j}\left(s,g\right),$$ (3)

$$\sigma^{j}\left(s,g\right)={\left(\overset{n}{\underset{i=1}{\sum }}\frac{w_{i}\left(s,g\right)}{\sigma_{i}^{j}\left(s,g\right)}\right)}^{-1}.$$ (4)

Here, $\mu_{i}^{j}$ and $\sigma_{i}^{j}$ represent the mean and variance of the i th skill network of the j th joint, respectively. The final desired position of the j th joint $\hat{q^{j}}$ is sampled from the composite Gaussian distribution $N\left(\mu^{j},\sigma^{j}\right)$ . The derivation of Equation 3 and Equation 4 can be found in Supplementary Material. It should be noted that we adopted a multiplicative model instead of an additive model in a hierarchical learning framework, such as a mixture of experts (MoE) (Jacobs et al., 1991), to avoid conflicting behaviors or blending artifacts caused by the sum of primitives, as reported by Peng et al. (2019).

##### 2.1.2.3 Control framework

The pre-trained single-skill policies, the gating network, and the composite multi-skill policy run at 25 Hz together generate desired joint positions that are tracked by joint-level PD controllers at 1,000 Hz. The PD controllers receive the desired joint positions $\hat{q}$ , measured joint positions q , and joint velocities $\dot{q}$ as input and output the joint torque commands $\tau =K_{p}\left(\hat{q}-q\right)+K_{d}\left(0-\dot{q}\right)$ .

##### 2.1.2.4 Reward design

For multi-skill learning, we design three groups of reward terms. The most important group of reward terms is related to goal tracking $r_{g}$ , another group is the reference foot-contact reward $r_{f}$ , and the last group includes the remaining reward terms in learning single locomotion skills $r_{e}$ . We set the overall reward r as the weighted sum of these terms as in Equation 5:

$$r=0.6r_{g}+0.2r_{f}+0.2r_{e}.$$ (5)

###### 2.1.2.4.1 Goal-tracking reward

Our goal-tracking reward consists of three terms. First, the relative position reward $r_{p_{g}}$ , which encourages minimization of the relative distance between the robot and the goal in the horizontal plane d≥0 as in Equation 6:

$$r_{p_{g}}=\varphi \left(p_{\mathrm{goal}}^{\mathrm{world}},p_{\mathrm{base}}^{\mathrm{world}},-0.74\right).$$ (6)

Second, the robot velocity reward $r_{v_{g}}$ is used together with the two other reward terms, encouraging the robot to track the goal as quickly as possible, although the relative position to the goal is dominant, as reflected in the reward weights given in Equation 7. It should be noted that the other reward terms, apart from the goal-tracking reward, also constrain the learned behaviors to be reasonable and feasible.

$$r_{v_{g}}=v^{2}.$$ (7)

The third term is the robot heading reward $r_{\phi_{g}}$ , which encourages alignment of the robot heading toward the goal as in Equation 8:

$$r_{\phi_{g}}=\varphi \left(u_{\mathrm{goal,base}}^{\mathrm{base}},\left[1,0,0\right],-2.35\right),$$ (8)

where $u_{\mathrm{goal,base}}^{\mathrm{base}}$ is the unit vector pointing from the robot base to the goal in the base frame. Our full goal-tracking reward is $r_{g}=r_{h_{z}}r_{\phi }\left(8r_{p_{g}}+4r_{v_{g}}+4r_{\phi_{g}}\right)$ , which increases the goal-tracking reward weight when the robot is closer to the nominal standing pose. $r_{h_{z}}$ and $r_{\phi }$ are the base height reward and base orientation reward, respectively, as shown in Table 1. The agent will prioritize these two rewards to ensure that the robot maintains the desired height and orientation in the initial stages and then progresses to maximize the goal-tracking reward.

###### 2.1.2.4.2 Reference foot-contact reward

Regarding the reference foot-contact reward in Table 1, learning different gaits requires different reference contact patterns. For multi-skill training, we activate different gaits according to the relative distance d between the goal and the robot, as described in the goal-tracking reward. Specifically, we use the trotting contact pattern as the reference to calculate this reward if $|d|<x_{1}$ , the bounding contact pattern if $x_{1}\leq |d|<x_{2}$ , and the gallop contact pattern if $|d|\geq x_{2}$ , where $x_{1}$ and $x_{2}$ are the gait switch criteria, which are discussed in Section 2.2 via an optimization loop outside the motor learning progress. In addition, it should be noted that for single skill policies, trotting has a similar velocity range as pacing and is a more common and stable gait. Therefore, when training our multi-skill policy, trotting was activated by the related reward terms when the goal was close rather than pacing. Nevertheless, technically, pacing can also be included for training a new multi-skill policy if needed.

### 2.2 Discovery of the skill-switching criteria

In this section, we propose setting up an optimization problem in the outer loop of the motor learning process to automatically discover the gait-switch criteria from trotting to bounding and from bounding to galloping. We use covariance matrix adaptation evolution strategy (CMA-ES) (Hansen, 2016), which is a derivative-free evolution strategy inspired by biological evolution for optimization. Here, we use the relative distance between the robot and the goal as the gait-switch criterion, as a proof of concept. We aim to find the gait-switch criteria $x_{1},x_{2}$ to maximize the sum of goal-tracking reward $r_{g}$ over each episode. The optimization problem is formulated as follows:

$$\begin{array}{ll} \arg\underset{x_{1},x_{2}}{\min} & \overset{N}{\underset{i=1}{\sum }}-r_{g}\left(x_{1},x_{2}\right)\\ \text{s.t.}\; & 0\leq x_{1}\leq 15,\\ & 0\leq x_{2}\leq 15,\\ & x_{1}<x_{2}. \end{array}$$

Here, $x_{1},x_{2}$ are the decision variables representing the relative distance between the robot and the goal in the horizontal plane to activate switching between trotting and bounding and between bounding and galloping gaits, respectively. N is the number of time steps in an episode.

It should be noted that our framework is not restricted to using relative distance as the gait-switch criterion. To demonstrate the effectiveness and generalizability of our proposed framework, we also use velocity as the gait-switch criterion in a velocity-tracking locomotion task, which can enrich our framework with more locomotion tasks and scenarios. Section 3.5.2 contains additional details.

## 3 Results

This section first introduces the experimental setup and then presents the optimization results for the gait-switch criteria. We then demonstrate that the proposed multi-skill policy achieves versatile locomotion gaits and their continuous transitions. Moreover, we showcase robust multi-skill locomotion in various test scenarios. Furthermore, our proposed framework illustrates its generalizability by acquiring multi-skill locomotion with two different formulations of gait-switch criteria: distance and velocity. Comprehensive ablation studies validate that our approach outperforms the baseline, with improved learning performance and more continuous gait transitions.

### 3.1 Experimental setup

#### 3.1.1 Multi-skill training setup

We sample 5,000 steps from the composite multi-skill policy for each training epoch, i.e., 20 episodes without early termination. Each episode lasts 10 s; the batch size is 128; the replay buffer size is 1e6; the learning rate is 3e-4; weight decay is 1e-6; the soft target update is 0.001; and the discount factor is 0.995 for fall recovery and 0.955 for locomotion gaits.

#### 3.1.2 Skill-switch criterion optimization setup

We set the initial gait-switch criteria as $x_{1}=2.0m$ and $x_{2}=5.0m$ to warmstart the optimization; the population size is 50, and σ=1.0m . The CMA-ES optimization runs for an iteration for every 20 iterations of inner-loop deep reinforcement learning.

#### 3.1.3 Goal trajectory setup

**FIGURE 4:**
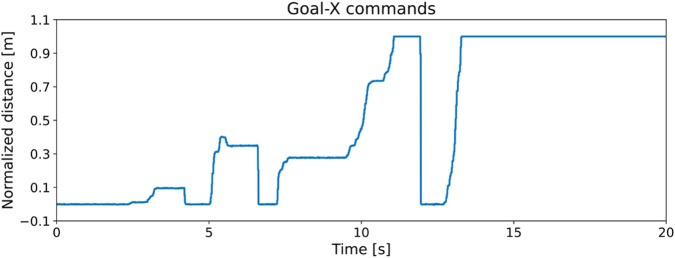
Normalized relative goal command in the robot heading frame is provided to encourage fall recovery, trotting, bounding, galloping, and their transitions.

We provide normalized relative goal distance in the x- and y-axes in the robot heading frame via a joystick in real-world tests to encourage the emergence of multiple dynamic skills and their transitions. An example goal trajectory is shown in Figure 4.

#### 3.1.4 Velocity estimation

During the deployment of multi-skill policies learned in simulation on real robots, sensing errors and uncertainties usually cause discrepancies between simulation and the real world. Unlike other state observations we selected for learning, base linear velocity cannot be obtained directly and needs to be estimated via leg kinematics or visual odometry, where the estimation results do not perform well during foot slipping or highly dynamic motions (Ji et al., 2022). Therefore, similar to Ji et al. (2022), we train a separate velocity estimator to obtain the estimation of unavailable or unreliable states given the sensory information of more reliable states. The input to the state estimator is 66-dimensional, including the gravity vector from roll and pitch measurements from the IMU, a two-step history of the gravity vector, the base angular velocity from the IMU, a two-step history of the base angular velocity, joint positions, a two-step history of joint positions, and joint velocity from motor encoders. The output is three-dimensional estimated base linear velocity. The estimator network is composed of two hidden layers, each with 256 neurons and a ReLU activation function. After we obtain the locomotion policies in simulation, we collect 215,000 pairs of input–output for training the estimator network via supervised learning. We use mean squared error loss (comparing ground-truth velocity with the velocity estimated by the neural network) for training, with a learning rate of 0.001, a weight decay of 0.0005, and a batch size of 1,024.

### 3.2 Optimized skill-switch criteria

**FIGURE 5:**
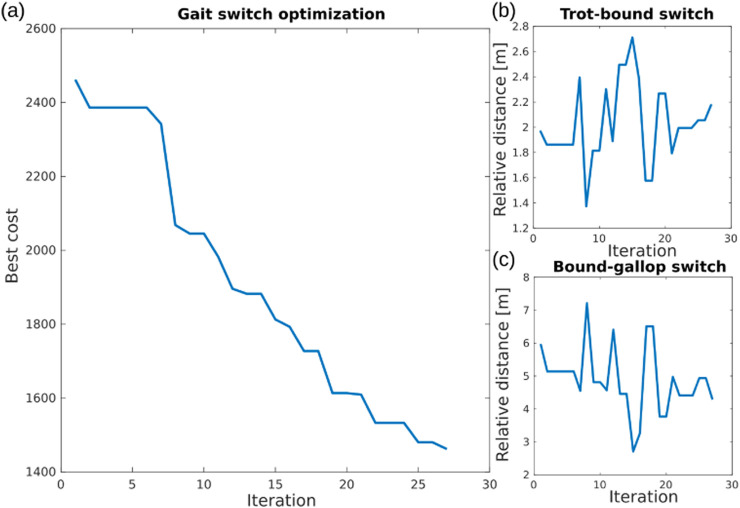
Results of CMA-ES optimization for gait-switch criteria in learning multi-skill locomotion. **(a)** Best cost during CMA-ES optimization. **(b)** Optimized relative distance for switching from trotting to bounding. **(c)** Optimized relative distance for switching from bounding to galloping.

Figure 5 shows that the best cost value keeps decreasing and reaches a local minimum value after 27 iterations of optimization, where the corresponding trot–bound and bound–gallop switch criteria are $x_{1}=2.2m$ and $x_{2}=4.3m$ , respectively. It should be noted that the optimized gait-switch criteria are not exactly where the gait transitions occur in practice since they are only incorporated into reward functions. Instead, the gait transitions during multi-skill locomotion are naturally learned via the optimized gait-switch criteria.

### 3.3 Multi-skill locomotion with continuous skill transitions

**FIGURE 6:**
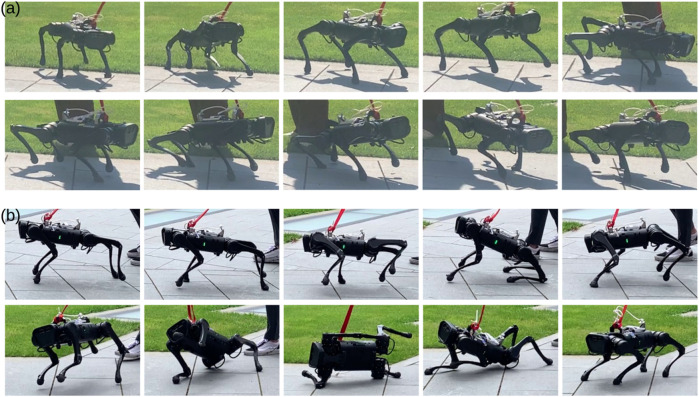
Comparison of multi-skill locomotion using our proposed approach and baseline. **(a)** Learned multi-skill locomotion by following goal trajectories in Figure 4. **(b)** Baseline approach by manually switching between learned single skills. The robot failed after a discrete switch from bounding to galloping.

With the learned multi-skill locomotion policy, the robot is able to demonstrate trotting, bounding, galloping, and prompt fall recovery whenever necessary, as shown in Figure 6a and the Supplementary Video S1. The corresponding goal commands provided via joystick are shown in Figure 4. Here, we report only the experimental results obtained in the real world. The video shows more simulation and robustness tests.

#### 3.3.1 Estimated speed

**FIGURE 7:**
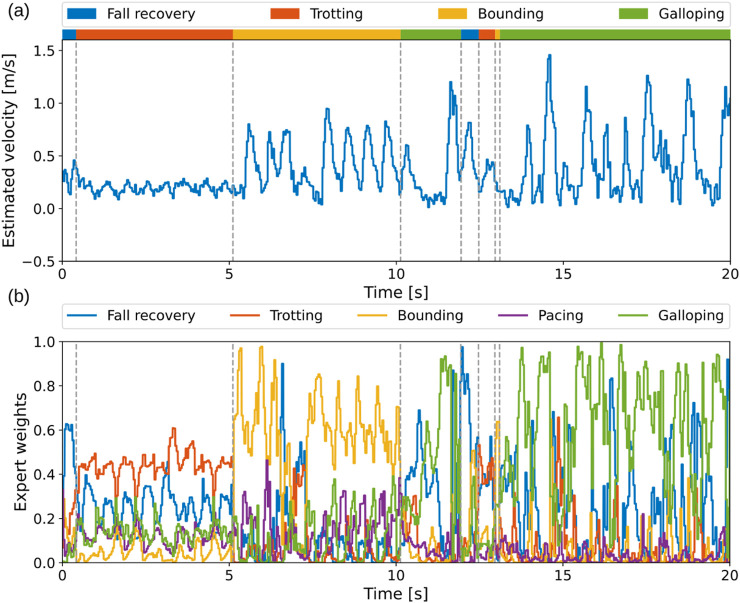
Versatile locomotion with continuous gait transitions on the real robot. **(a)** Estimated horizontal speed of the robot during multi-skill locomotion. **(b)** Expert weights showing that each motion utilizes all five experts, with one related expert being dominant.

We show the estimated speed in the horizontal plane of the robot heading frame for 20 seconds of the multi-skill locomotion (Figure 7a). The robot shows an increasing velocity for trotting, bounding, and galloping skills.

#### 3.3.2 Expert weights

**FIGURE 8:**
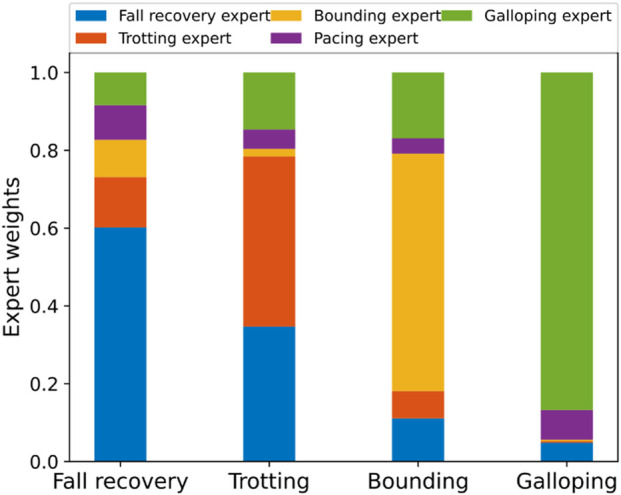
Composition of five skill primitives for fall recovery, trotting, bounding, and galloping during multi-skill locomotion at 0.2 s, 2.5 s, 8.0 s, and 18.0 s, respectively.

Figure 7b shows the weights for each single-skill policy generated by the gating network correspondingly. The fall recovery expert dominates during the recovery motion. For the three locomotion gaits exhibited, each corresponding expert has the largest weight among all. However, compared to bounding and galloping gaits, where the corresponding expert dominates, the trotting expert co-acts more together with the other experts during trotting, contributing to the synthesized policy. Furthermore, to obtain a detailed view of the influence of each expert in different quadruped locomotion skills, we visualized the weight of each expert for the four demonstrated skills at different time-steps. Figure 8 clearly shows the composition of single-skill policies for each motion, with each motion utilizing all five single skills.

#### 3.3.3 Euler angles

**FIGURE 9:**
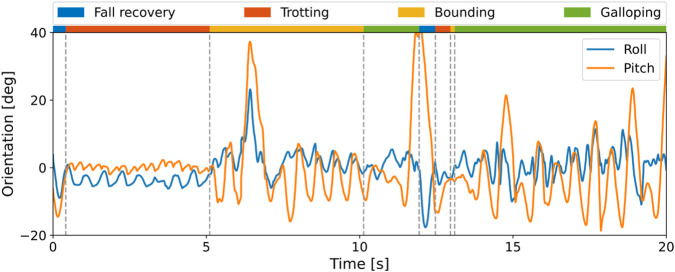
Roll and pitch angles during multi-skill locomotion in the real world.

The corresponding roll and pitch angles are shown in Figure 9. When the robot encountered falls, the roll and pitch angles increased at first and returned to the normal range during fall recovery. In other cases, these two Euler angles have clear cyclic patterns. Moreover, the magnitude of the Euler angles increases as the robot progresses from trotting to bounding to galloping, indicating that the motion becomes more dynamic.

### 3.4 Robustness tests

**FIGURE 10:**
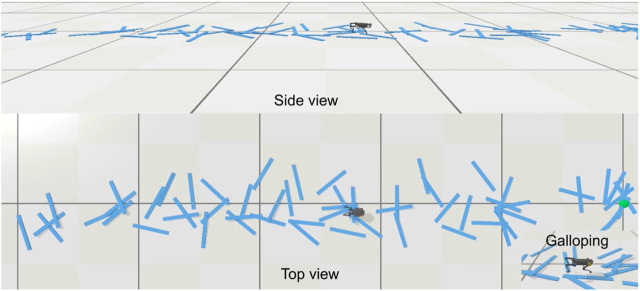
Learned multi-skill policy enabling the robot to traverse the terrain with random obstacles with natural gait transitions in physics simulation. The robot can perform highly dynamic galloping gait on a rough terrain.

Supplementary Video S1 showcases the robustness tests of the learned multi-skill policy in physics simulation, including (1) successfully traversing terrains with random obstacles (Figure 10), (2) locomoting with varying body mass, and (3) locomoting with input noise. Please refer to the video for the robot in action.

### 3.5 Ablation studies

#### 3.5.1 Distance as the gait-switch criterion: discrete switch vs. our approach

We compare our proposed multi-skill learning and optimization approach with the baseline approach, i.e., manual switching between different skill primitives. For the single skills, trotting and bounding were learned with a fixed desired velocity, while galloping was learned by maximizing velocity. After multi-skill learning with fixed parameters of each expert network, trotting motion is synthesized by the gating network at a lower speed range, and galloping motion is synthesized in a more dynamically feasible pattern. The snapshots in Figure 6 and Supplementary Video S1 contain more details.

**FIGURE 11:**
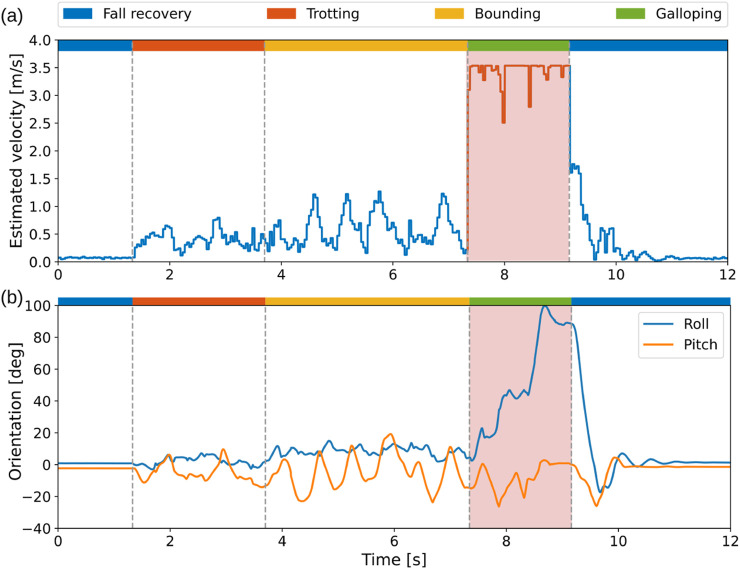
Performance of the baseline approach by manually switching from fall recovery to trotting to bounding in the real world. Robot failed to switch to galloping from bounding (red shaded areas) but was then able to perform a successful recovery from failure to standing still. **(a)** Estimated horizontal speed. **(b)** Roll and pitch angles.

For the baseline approach, the robot failed when manually switching from bounding to galloping, sometimes causing automatic shutdown due to the power protection for Unitree robots. In cases where failure occurs without triggering power protection, we can manually activate the fall-recovery skill, after which the robot can recover from the failure and return to a standing state. However, to resume locomotion, it requires another discrete switch from standing to trotting, causing further instability issues. In contrast, our multi-skill policy can directly transition from failure to trotting without the intermediate phase in a dynamic fashion. When discretely switching from trotting to bounding at an improper gait phase, the knee joints of the rear legs may reach very close to the ground, or the front legs may lift very high in the following several time-steps. As shown in Figure 11, manual switch caused dynamic instability, such as abrupt changes in estimated velocity. In contrast, our approach enables a smoother and continuous gait transition in real-world deployment.

#### 3.5.2 Velocity as the gait-switch criterion

**FIGURE 12:**
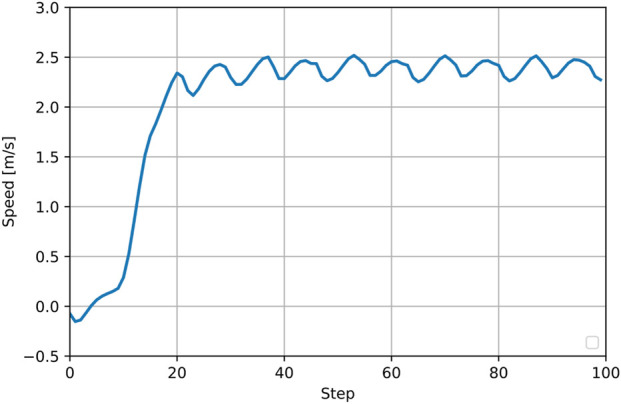
Example of the speed profile using velocity as the gait-switch criterion.

In addition to robot’s distance to the goal, our framework can also use other physical quantities as the gait-switch criteria, such as velocity. We formulate this in a velocity-tracking task setting. The velocity command to follow in the x-direction is sampled from the range of 0∼3m/s during training. The goal-tracking reward terms are replaced with velocity-tracking reward terms. The reference foot-contact reward is segmented by the desired velocities for gait switching. For the outer optimization loop, the cost function is changed to minimize CoT as in Equation 9:

$$CoT=\frac{\sum_{i}^{12}\max\left(\tau_{i}\omega_{i}+\alpha \tau_{i}^{2},\;0\right)}{mg\|v\|},$$ (9)

where $\tau_{i}$ and $\omega_{i}$ are the joint torque and joint velocity of the i th joint, respectively; m is the robot mass; g is gravity; ‖v‖ is the velocity norm; and α=0.3 , which is robot-specific. We set the initial gait-switch criteria as $v_{1}=0.6m/s$ and $v_{2}=1.2m/s$ to warmstart optimization, with a population size of 50 and σ=0.2m/s . The CMA-ES optimization runs for an iteration for every 20 iterations of inner-loop deep reinforcement learning. Figure 12 shows the smooth velocity curve when tracking a velocity command of 2.5m/s . In the following sections, we perform ablation studies on various design choices based on this implementation. In all subsequent ablation studies, the metric used to evaluate and benchmark learning performance is the training reward, and each training curve shows the mean and standard deviation of the reward across three training trials, averaged using a sliding window of 20 steps for visualization.

##### 3.5.2.1 Outer optimization loop

**FIGURE 13:**
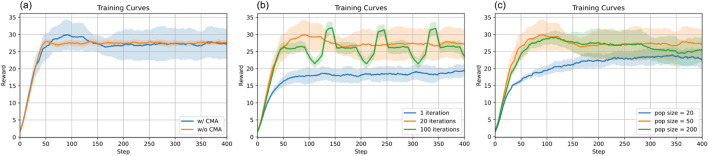
Ablation of gait-switch criterion optimization. **(a)** Training curves with and without the gait-switch criterion optimization. **(b)** Training curves of optimization update intervals with respect to RL agent updates. **(c)** Training curves with different population sizes in CMA-ES for gait-switch criterion optimization.

We removed the outer optimization loop and retained only the hierarchical RL component of our framework and compared its learning performance with that of the full framework including the optimization loop. Figure 13a shows that our framework yields a higher reward than the baseline without the outer optimization loop.

##### 3.5.2.2 Ablation on optimization parameters

Here, we ablate two important parameters in CMA-ES optimization. (1) Optimization update intervals with respect to RL updates. In our framework, we run one iteration of CMA-ES optimization for every 20 iterations of RL updates, which is noted as 1/20. In this ablation study, we compare this parameter 1/20 with 1/1 and 1/100. From Figure 13b, we find that optimizing one iteration per RL iteration results in a final reward that is far from optimal. Compared with one optimization per 100 RL updates, our implementation converges to a higher reward earlier. (2) Population size. Population size is the number of candidate solutions sampled for each generation in CMA-ES. In our implementation, we set this parameter to 50. Here, we compare it with 20 and 200. From Figure 13c, we find that setting the population size as 20 is not sufficient to converge to a high reward. Compared to 100, our chosen population size converges to a slightly higher reward more quickly.

##### 3.5.2.3 Ablation on hierarchical RL frameworks

**FIGURE 14:**
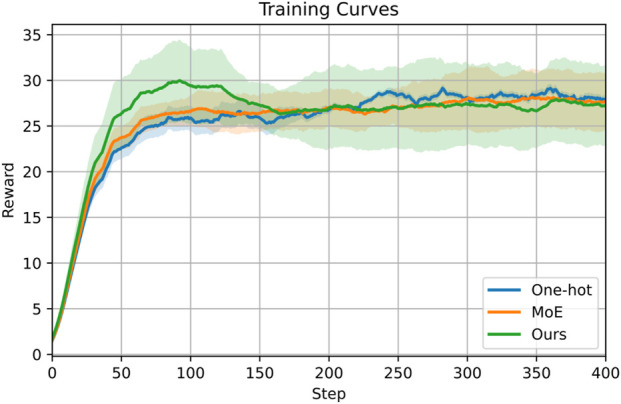
Ablation of hierarchical RL frameworks combining multiple low-level individual skills by one-hot vector, mixture of experts, and ours.

Different hierarchical RL frameworks modulate and fuse low-level individual skills in different ways. Our framework uses a high-level gating network to modulate individual skills by fusing their Gaussian distributions. Here, we compare this implementation with two commonly used approaches. Note that the outer optimization loop is included in this comparison. (1) One-hot vector: The gating network learns to generate a one-hot vector to select one low-level skill per step. (2) MoE: The gating network learns to generate weights for the output of each expert that are summed as the final output. Figure 14 shows that MoE converges faster than the one-hot vector. Furthermore, our implementation of the hierarchical RL framework achieves a faster convergence rate and a higher reward than the one-hot vector and MoE baselines.

## 4 Discussion

This research developed a hierarchical learning and optimization framework to achieve multi-skill locomotion with optimized gait-switch criteria without the need for any reference trajectories or expert demonstrations. The robot demonstrates continuous gait transitions among trotting, bounding, and galloping skills as locomotion speed increases. Our learned multi-skill policy can also incorporate the fall recovery skill, which enables the robot to recover promptly and resume locomotion whenever it becomes unstable or falls during different gaits. Thus, the robot requires less human intervention to operate autonomously in a remote working space, enabling versatile applications. Compared with the existing end-to-end learning framework using a single policy, our hierarchical framework is bio-inspired and more efficient in fine-tuning for various tasks since it does not require learning from scratch to adapt to new tasks. Moreover, by optimizing gait-switch criteria as motor learning progresses, we avoid manually specifying the criteria with biased human knowledge distillation. The formulation can easily be adapted and generalized to different tasks or scenarios by customizing the cost function. It should be noted that the three-segment reward terms to encourage different foot-contact patterns based on the robot’s distance to the goal (gait switch criteria) are discrete; however, the actual gait transition does not occur abruptly. This is because the distance we optimize is the desired distance to switch and not the actual distance, and the actual distance is also determined during learning by the other reward terms regarding smooth and stable locomotion.

One limitation of our approach is that we found that sim-to-real discrepancies still exist in the galloping motion. In the simulation, the galloping motion is more stable without any failures. In the real world, we cannot ensure a 100% success rate of galloping for very long periods. This can be due to various reasons. Compared to other locomotion skills, galloping is inherently a very unstable locomotion skill, since only one foot is in contact with the ground at one time. Slight sim-to-real discrepancies can cause huge differences and even failures, such as deformable foot pads on the Unitree A1 robot, ground friction, and velocity estimation for out-of-the-distribution motion. Another reason is that the goal commands used in real-world tests differ from those in the simulation. For training in simulation, we provide the goal position directly in the world frame, while in the real world, due to the lack of body-position feedback, we provide normalized relative goal distance in the robot heading frame via joystick; thus, it is not possible to reproduce the same goal commands as in simulation via joystick.

For future work, we plan to further resolve the sim-to-real gap in galloping motion. We would also like to analyze the scalability of the proposed framework to more skills and more complex tasks as it would need more reward engineering. Furthermore, since our proposed approach can generate multi-skill locomotion data without any reference, one interesting application of our proposed framework would be preparing datasets for the training and fine-tuning of generalist policies for legged robots, such as diffusion models or OpenVLA (Kim et al., 2025).

## Data Availability

The raw data supporting the conclusions of this article will be made available by the authors, without undue reservation.
