# Supplementary material for: Discovery of skill-switching criteria for learning agile quadruped locomotion
Source: Front Robot AI. 2026 Feb 18;13:1697159. doi: 10.3389/frobt.2026.1697159 (PMC12957656; doi:10.3389/frobt.2026.1697159)
Supplement: Supplementary file 1 [file Presentation1.pdf]

Supplementary Material for

Discovery of skill-switching criteria for learning agile quadruped locomotion

Wanming Yu, Fernando Acero, Vassil Atanassov, Chuanyu Yang,

Ioannis Havoutis, Dimitrios Kanoulas, and Zhibin Li

1 DERIVATION OF WEIGHTED PRODUCTS OF n GAUSSIAN PDFS

Given n pre-trained single-skill Gaussian policies:

$$\pi_i(a) = \mathcal{N}(a \mid \mu_i, \sigma_i) = \frac{1}{\sqrt{2\pi\sigma_i}} \exp\left[-\frac{(a-\mu_i^2)}{2\sigma_i}\right]$$

The composite multi-skill policy is defined as the weighted multiplicative composition:

$$\pi(a) \propto \prod_{i=1}^{n} \pi_i(a)^{w_i}, \quad w_i \geq 0$$

Ignoring normalization constants, we have:

$$\begin{aligned}\pi(a) &\propto \prod_{i=1}^{n} \exp\left(-\frac{w_i(a-\mu_i)^2}{2\sigma_i}\right) \\ &= \exp\left[-\frac{1}{2}\sum_{i=1}^{n}\frac{w_i}{\sigma_i}(a-\mu_i^2)\right]\end{aligned}$$

Expanding the quadratic form

$$\begin{aligned}\sum_{i=1}^{n}\frac{w_i}{\sigma_i}(a-\mu_i)^2 &= \sum_{i=1}^{n}\frac{w_i}{\sigma_i}\left(a^2 - 2a\mu_i + \mu_i^2\right) \\ &= a^2 \underbrace{\sum_{i=1}^{n}\frac{w_i}{\sigma_i}}_{\tau} - 2a \underbrace{\sum_{i=1}^{n}\frac{w_i}{\sigma_i}\mu_i}_{\xi} + \underbrace{\sum_{i=1}^{n}\frac{w_i}{\sigma_i}\mu_i^2}_{C}\end{aligned}$$

Thus the exponent becomes:

$$-\frac{1}{2}\left[\tau a^2 - 2\xi a + C\right]$$

Factor τ from the first two terms:

$$\tau a^2 - 2\xi a = \tau\left(a^2 - 2\frac{\xi}{\tau}a\right)$$

Complete the square:

$$\begin{aligned} a^2 - 2\frac{\xi}{\tau}a &= \left(a^2 - 2\frac{\xi}{\tau}a + \left(\frac{\xi}{\tau}\right)^2\right) - \left(\frac{\xi}{\tau}\right)^2 \\ &= \left(a - \frac{\xi}{\tau}\right)^2 - \left(\frac{\xi}{\tau}\right)^2 \end{aligned}$$

Substituting back:

$$\begin{aligned} \tau a^2 - 2\xi a &= \tau\left[\left(a - \frac{\xi}{\tau}\right)^2 - \left(\frac{\xi}{\tau}\right)^2\right] \\ &= \tau\left(a - \frac{\xi}{\tau}\right)^2 - \frac{\xi^2}{\tau} \end{aligned}$$

The full exponent becomes:

$$\begin{aligned} &-\frac{1}{2}\left[\tau\left(a - \frac{\xi}{\tau}\right)^2 - \frac{\xi^2}{\tau} + C\right] \\ &= -\frac{1}{2}\tau\left(a - \frac{\xi}{\tau}\right)^2 + \frac{1}{2}\left(\frac{\xi^2}{\tau} - C\right) \end{aligned}$$

The second term $\frac{1}{2}\left(\frac{\xi^2}{\tau} - C\right)$ is constant with respect to a. The remaining term is quadratic in a with mean and precision:

$$\mu = \frac{\xi}{\tau} = \frac{\sum_{i=1}^{n} \frac{w_i}{\sigma_i}\mu_i}{\sum_{i=1}^{n} \frac{w_i}{\sigma_i}}$$

$$\sigma = \frac{1}{\tau} = \left(\sum_{i=1}^{n} \frac{w_i}{\sigma_i}\right)^{-1}$$

2 REAL-TIME INFERENCE

Our proposed hierarchical neural network controller runs at 25 Hz. At each control step, a complete inference consists of: (1) a forward pass of a high-level fully-connected neural network, (2) forward pass of five low-level fully-connected neural networks, (3) analytical Gaussian fusion using Eq. 3 and 4, and (4) sampling from the fused Gaussian.

Among the above four steps, the dominant in inference is the matrix multiplications and additions in neural network inference and the rest is negligible. The computational complexity of a neural network with layer sizes $\{h_0, h_1, ..., h_L\}$ is $\mathcal{O}\left(\sum_{l=0}^{L-1} 2h_l\, h_{l+1}\right)$, which leads to constant-time inference per control step. In our setting, our controller roughly requires 921k FLOPs (total number of floating-point operations). The theoretical peak of NVIDIA Jetson TX2 used on the robot is 1.33 TFLOPS. The inference time on the control board would be negligible compared to our control loop at 40 ms. That is, real-time control can be achieved with our proposed controllers.
